# Pyemia

**Published:** 1875-04

**Authors:** E. C. White

**Affiliations:** La Grange


### PYEMIA.

BY E. C. WHITE, OF LA GRANGE.

[*Read before the N. E. Indiana Medical Society, at Ligonier, December* 30*th*, 1873.]

What is pyemia? It is defined as the development of multiple abcesses in some of the internal organs. The term is a corruption of the term pyohæmia, and is synonymous with ichorrhæmia—a term more recently introduced in pathological nomenclature. The literal meaning of the term pynemia is an alteration or poisoning by pus.

The exciting causes of pyonemia, or blood-poisoning, are numerous; any cause producing a severe shock to the system, such as excessive loss of blood, amputation of the larger limbs, gun-shot wounds of the head or larger articulations, gun-shot fractures, and the severe forms of railroad accidents. It has supervened in erysipelas, and may occur with lying-in women.

Gross says, to it may be attributed much of the mortality occurring in the puerperal state. As found in army hospital practice, it, perhaps, was more frequently found following gun-shot wounds of the more important articulation, and amputations of the thigh. Its approach is insidious and stealthy, the wound or stump to all appearances doing well and healing kindly; the first indications of trouble being a severe rigor, followed by flashes of heat, which are quickly followed by a rapid, feeble and variable pulse, and a dry, dark tongue. The rigors coming on at intervals of from two to twenty-four hours, alternating with exacerbations of febrile excitement. The appetite soon fails, and the system soon succumbs to the poison. The entire body soon presents a general ichtoroid appearance—the tongue becoming darker, red at the tip and edges, with an accumulation of sordies upon the lips, gums and teeth. Frequently, the patient sinks into a state of coma from which he never awakes. But, in some instances, full consciousness is retained until almost the last. In short, there is a general typhoid condition.

The question naturally arises, Is pus absorbed from the seat of injury? One class of pathologists contend that it is; but physiology teaches us that healthy pus cannot be absorbed and pass through the capillaries into the circulation; and, although they assert that they have seen the pus globule in the blood, doubtless they have taken the white corpuscles of the blood for pus globules, between which it is difficult, if not impossible, to distinguish : nor is it probable that pus is taken up by the open mouths of the cut veins, for they are glued together and entirely closed before suppuration has taken place. I can conceive of no instance in which this might occur, except an abscess be opened in the immediate neighborhood of one of the larger veins during an operation. But may it not be that disintegrated, saneous pus is absolutely poisoning the blood, and disposing to the formation of multiple abscesses found in the several organs, or inflammation may be excited in the coats of the injured veins, and this phlebitis be, in fact, the exciting cause—the inflammatory process going on to the stage of suppuration—and pus thus carried into the circulation, to become points of irritation wherever deposited in the net-work of the capillaries, thereby exciting inflammation in its parts, resulting in suppuration and the formation of multiple abscesses? I am, however, of opinion that every instance wherein pyemia arises, the pus will be found saneous, icherous and disintegrated. That it never supervenes in cases where the pus is healthy and well elaborated—at least, not in such cases, if free vent is given the discharge, so there can be no bagging or burrowing of pus among the muscles. If this burrowing be allowed, the pus must become unhealthy in character, disintegrated, and, of course, liable to absorption ; and every hour it is allowed to remain but enhances the danger ; and if once embedded among the muscles is very difficult to remove so entirely as to have the patient in a condition of safety.

The post-mortem appearances, as might be expected, are numerous and interesting, the most common being the purulent deposits, or multiple abscesses. These are found most frequently in the lungs and liver ; next in the spleen ; less frequently in the heart, brain and kidneys. The deposits may take place in the pelvic and peritoneal cavities, and have been found in the prostrate gland. The number of these multiple abscesses vary widely, from five or six to many hundreds. Gross mentions a case in which there was a thousand. I have seen cases where there were several hundred studding the lungs. The size varies with the number, being smaller when very numerous, and large when few in number. In size they were from a millet-seed to a good-sized orange; and, when numerous, often become confluent. The contents of these abscesses are not usually of the character of well-elaborate pus, but is grayish in color, having somewhat the appearance of the gray matter of the brain ; and, in consistency, is frequently similar to partially softened tubercular matter. I have observed, in every case I have examined, the uniformity of pleuritic adhesions—sometimes evidently of recent origin over one lung, and over the other so fine as to be almost impossible to separate it without tearing the substance of the lung; and I think the abscesses, as a rule, were more numerous in the lung the most firmly adherent. Pus is also found intermingled with fibrinous clots. The cadaver, I believe, always presents an ichtoroid appearance; the conjunctivæ deeply colored, and the skin being of nearly a golden yellow, tinged with green. This ichtoroid appearance is more prominent after than previous to death. The blood is found to be thin and aqueous, evidently robbed of its coloring-matter, so much as scarcely to stain a cloth, and seemingly having lost all plasticity and power of coagulation.

The prognosis in pyemia is extremely unfavorable, recovery being rare, and, in these, the convalescence is very slow and tedious, the least exposure or indiscretion in diet causing a relapse. The system is also more than usually liable to attacks from other diseases, to which it rapidly yields in its already debilitated condition.

The treatment of pyemia is sustaining, tonic and stimulating.—Beef-tea, brandy in milk punch, wines, wine-whey, or the more diffusible stimulants, as carbonate of ammonia, liquor ammonia, quinine, iron, etc. Quinine, when given, should be in full doses—not less than x. grs. at once—combined with sufficient morphia to produce a full anodyne effect. To give it in small, frequently-repeated doses but teases the system and aggravates the symptoms. By putting the patient at once under the influence of the remedy, rest and quietude are obtained, and opportunity given Nature to rally from the depressing influence of the poison. I think the preparatives of iron to be preferred are, the chlorinated tincture and the sulphate—not only for their tonic but antiseptic powers. The utmost cleanliness should be observed, the wound being frequently and thoroughly cleansed, not only giving free vent to all discharges, but the parts should be thoroughly washed out, or even by means of a syringe, if necessary for their removal. The clothing and bedding should be changed at least daily, and the fresh clothing well aired and dried before use. All dressings should be removed at once from the bed-side. Free and full ventilation is absolutely essential. The diet should be light, nutritious and easily digested.—Beef-tea, light boiled eggs, milk, chicken-broth, meat-broths, of which mutton is perhaps the best, and jellies. The thirst is usuallly intense and urgent, but the patient should avoid large draughts of water, for it overloads the stomach and, no doubt, interferes materially with the action of remedies, and obstructs the free digestion of the little nourishment which the patient is able to take. Great relief may be obtained by swallowing small pieces of ice; even holding them in the mouth and allowing them to dissolve there, will often give decided relief.—*Indiana Journal of Medicine.*

